# Multi-CAR: a tool of contig scaffolding using multiple references

**DOI:** 10.1186/s12859-016-1328-7

**Published:** 2016-12-23

**Authors:** Kun-Tze Chen, Cheih-Jung Chen, Hsin-Ting Shen, Chia-Liang Liu, Shang-Hao Huang, Chin Lung Lu

**Affiliations:** Department of Computer Science, National Tsing Hua University, Hsinchu, 30013 Taiwan

**Keywords:** Bioinformatics, Next-generation sequencing, Contigs, Scaffolding, Multiple references

## Abstract

**Background:**

A draft genome assembled by current next-generation sequencing techniques from short reads is just a collection of contigs, whose relative positions and orientations along the genome being sequenced are unknown. To further obtain its complete sequence, a contig scaffolding process is usually applied to order and orient the contigs in the draft genome. Although several single reference-based scaffolding tools have been proposed, they may produce erroneous scaffolds if there are rearrangements between the target and reference genomes or their phylogenetic relationship is distant. This may suggest that a single reference genome may not be sufficient to produce correct scaffolds of a draft genome.

**Results:**

In this study, we design a simple heuristic method to further revise our single reference-based scaffolding tool CAR into a new one called Multi-CAR such that it can utilize multiple complete genomes of related organisms as references to more accurately order and orient the contigs of a draft genome. In practical usage, our Multi-CAR does not require prior knowledge concerning phylogenetic relationships among the draft and reference genomes and libraries of paired-end reads. To validate Multi-CAR, we have tested it on a real dataset composed of several prokaryotic genomes and also compared its accuracy performance with other multiple reference-based scaffolding tools Ragout and MeDuSa. Our experimental results have finally shown that Multi-CAR indeed outperforms Ragout and MeDuSa in terms of sensitivity, precision, genome coverage, scaffold number and scaffold N50 size.

**Conclusions:**

Multi-CAR serves as an efficient tool that can more accurately order and orient the contigs of a draft genome based on multiple reference genomes. The web server of Multi-CAR is freely available at http://genome.cs.nthu.edu.tw/Multi-CAR/.

## Background

In the past decade, the techniques of next-generation sequencing (NGS) have advanced greatly so that an increasing number of genome sequences can be produced rapidly at a moderate cost [1]. Nevertheless, most of the genomes sequenced by currently NGS techniques are just *draft* (or *unfinished*) genomes with collections of independent contigs whose relative positions and orientations along the genome being sequenced are unknown [2]. To address this issue, a process called *scaffolding* is then used to order and orient the contigs in a draft genome [3]. After that, the subsequent finishing process utilizes a so-called primer walking technique to closing the gaps between ordered and oriented contigs [4]. Currently, however, the primer walking procedure is still expensive and work-intensive. Therefore, the accuracy of the scaffolding process can be very helpful to obtain a complete genome of an organism in the finishing process, because given *n* ordered and oriented contigs, only $\mathcal {O}(n)$, instead of $\mathcal {O}(n^{2})$, primer walking procedures are needed to close the gaps between them, greatly reducing the cost and time for completely sequencing genomes.

Actually, in addition to paired-end read mapping approaches [5, 6], resequencing is another commonly used approach in the scaffolding process [7]. Usually, the resequencing approaches require a complete genome of a related organism to serve as a reference. Basically, given a target draft genome and its reference genome, the resequencing methods first map the contigs onto the reference genome and then infer the ordering and orientations of the contigs according to their positions on the reference genome. Currently, several scaffolding tools based on the resequencing approach have been proposed. However, most of them use only one reference genome to derive the order and orientations of contigs, such as OSLay [8], ABACAS [9], Mauve Aligner [10], fillScaffolds [11], r2cat [12], SIS [13] and CAR [14]. As evaluated in our previous study [14], CAR we implemented based on a rearrangement-based algorithm [15] has a better performance among all these single reference-based scaffolding tools in terms of average sensitivity, precision and genome coverage. However, all these single reference-based scaffolding tools may produce erroneous scaffolds (i.e., ordered and oriented contigs) if there are rearrangements between the target and reference genomes or their phylogenetic relationship is distant. This may suggest that a single reference genome may not be sufficient to produce correct scaffolds of a target draft genome.

Ragout [16] and MeDuSa [17] are recently developed scaffolding tools based on the resequencing approach using multiple reference genomes. Given a target of draft genome, multiple reference genomes, and a phylogenetic tree of them, Ragout represents all the target and reference genomes as sequences of synteny blocks (or lists of signed numbers). It then creates a so-called incomplete multi-color breakpoint graph, in which vertices represent the ends of synteny blocks and edges denote adjacencies of two synteny blocks occurring in the target and reference genomes. In addition, each edge is colored by using the color of the genome in which its corresponding adjacency occurs. Basically, the target genome is fragmented into contigs and hence some adjacencies of synteny blocks in the target genome are missing. Next, Ragout tries to recover these missing adjacencies by utilizing other existing adjacencies from the reference genomes. In this process, it requires to calculate the parsimony costs of all possible missing adjacencies by solving a so-called half-breakpoint state parsimony problem on the given phylogenetic tree, which is already known to be NP-hard. Hence, Ragout instead utilizes a heuristic method to obtain the approximate parsimony costs of all possible missing adjacencies. After that, it finds a perfect matching with minimum cost from a graph constructed by all possible missing adjacencies to order and orient the contigs of the target genome. In fact, Ragout repeats the above procedure several times with different synteny block sizes and then combines the scaffolds returned in all the iterations into a single set of scaffolds. In addition, Ragout performs a refinement step to insert some small and repetitive contigs back to the resulting scaffolds.

As for MeDuSa, it constructs a so-called scaffolding graph from the given target and reference genomes (without requiring a given phylogenetic tree), in which vertices represent contigs in the target genome and weighted edges denote adjacencies of two contigs if they can be mapped to the reference genomes, where the weight of an edge indicates how many reference genomes support the existence of such contig adjacency. Next, since a path in the scaffolding graph corresponds to an order of some contigs, MeDuSa tries to find a path cover with maximum weight from the scaffolding graph. However, the path cover problem is known as NP-hard. In the above process, MeDuSa hence utilizes a 2-approximation algorithm to find an approximate path cover from the scaffolding graph. Finally, MeDuSa applies a majority rule to determine the orientations of contigs on each path of the approximate path cover.

In this study, we revise our single reference-based scaffolding tool CAR [14] into a new web server called Multi-CAR (multiple-reference version of CAR) by a simple heuristic method such that it can utilize multiple complete genomes of related organisms as references to more accurately order and orient the contigs of a draft genome. Like MeDuSa, our Multi-CAR does not require prior knowledge concerning phylogenetic relationships among target and reference genomes and libraries of paired-end reads. However, in contrast to Ragout and MeDuSa, both attempting to solve an NP-hard problem, the algorithm behind our Multi-CAR involves only polynomially solvable problems. To validate Multi-CAR, we have tested it on a real dataset composed of several prokaryotic genomes and also compared its performance with Ragout and MeDuSa. As a consequence, our experimental results have shown that Multi-CAR indeed performs better than Ragout and MeDuSa in terms of many metrics like sensitivity, precision, genome coverage, scaffold number and scaffold N50 size.

## Methods

### Overview of CAR

In the study of CAR [14], we formulated the single reference-based scaffolding problem as follows: Given a target genome *π* with a set of contigs and a reference genome *σ*, the goal of the problem is to order and orient the contigs of the target genome in a way that minimizes the rearrangement distance between the ordered and oriented target genome and the reference genome. Basically, there are many rearrangement operations to measure the distance between two genomes. In CAR, we used reversals and block-interchanges with weight ratio 1:2 to measure such rearrangement distance and moreover utilized the techniques of permutation groups in algebra to compute it. To apply the permutation groups on *π* and *σ*, we needed to represent them as two permutations of *n* signed integers between 1 and *n*, where each integer denotes a conserved genetic marker between *π* and *σ* and its sign represents the strandedness of the corresponding genetic marker. For this purpose, we used the program NUCmer or PROmer from MUMmer’s package [18] to detect conserved genetic markers between *π* and *σ*. Note that in this process, NUCmer was performed on nucleotide sequences of *π* and *σ*, while PROmer was performed on amino acid sequences of *π* and *σ* translated from their nucleotide sequences in all six reading frames. After that, we applied an efficient algorithm we designed based on the permutation groups in [15] on the signed permutations of *π* and *σ* to order and orient the contigs of *π* according to the reference genome *σ*. Basically, we considered a contig as a linear chromosome and the job of scaffolding two contigs as a *fusion* of their corresponding chromosomes. Suppose that there are *m* contigs in *π*. Then our algorithm in [15] can find *m*−1 fusions to join these *m* contigs in *π* in linear time such that the resulting *π* has the minimum rearrangement distance from *σ*. We refer the reader to our paper [15] for the details of the above algorithm.

### Method of Multi-CAR

**Fig. 1:**
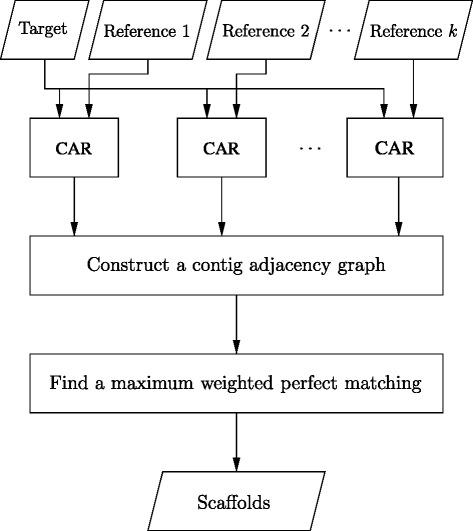
The procedure flowchart of multi-CAR

**Fig. 2:**
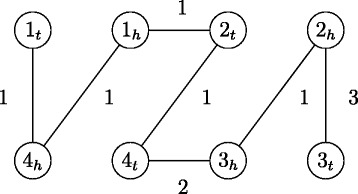
A contig adjacency graph constructed by four scaffolds *S*_1_=(+1,+2,+3), *S*_2_=(+2,+3,+4), *S*_3_=(−1,−4,−3,−2) and *S*_4_=(+1,−4,+2,−3), where the dummy edges with zero weight are omitted

The method we used to implement Multi-CAR is as follows (see Fig. 1 for its procedure flowchart). First, given a target genome *T*={1,2,…,*n*} with a set of *n* contigs and *k* references of complete genomes *R*_1_,*R*_2_,…,*R*_*k*_ with weights *W*_1_,*W*_2_,…,*W*_*k*_, respectively, we apply CAR to order and orient the contigs of the target genome based on each reference genome. Note that the output returned by CAR is a list of scaffolds, with each consisting of the ordered and oriented contigs. Basically, a contig *c*∈*T* represents an oriented linear sequence of DNA starting with a *tail* and ending with a *head*. The tail and head of *c* are also called *extremities* and denoted by *c*_*t*_ and *c*_*h*_, respectively, in this study. By reading the contigs of a scaffold in the left-to-right direction, if the tail of a contig *c* precedes its head, then we write this contig as +*c* in the scaffold; otherwise, we write it as −*c*. Second, we utilize all the scaffolds returned by CAR to build a *contig adjacency graph* *G*=(*V,E*) as follows. For each contig *c*∈*T*, there are two vertices *c*_*t*_ and *c*_*h*_ in *V*, that is, *V*={*c*_*t*_,*c*_*h*_|*c*∈*T*}. In *E*, there is an edge to connect two vertices if they are adjacent extremities from two different contigs that are ordered consecutively in a scaffold returned by applying CAR to *T* and *R*_*i*_, where 1≤*i*≤*k* (i.e., the reference genome *R*_*i*_ supports that these two contigs should be ordered and linked together in the target genome). If there are multiple reference genomes to support this edge connection, then this edge will be assigned a weight that equals to the sum of the weights of the supporting reference genomes. In addition, to guarantee the existence of a perfect matching in *G*, we add a dummy edge with zero weight into *G* to connect any two vertices that are from two different contigs and not supported to be connected by any reference genome. Note that in *G*, there is no edge between any two vertices that come from the same contig. For example, suppose that *S*_1_=(+1,+2,+3), *S*_2_=(+2,+3,+4), *S*_3_=(−1,−4,−3,−2) and *S*_4_=(+1,−4,+2,−3) are the scaffolding results respectively obtained by applying CAR on a target genome of four contigs *T*={1,2,3,4} and four reference genomes *R*_1_,*R*_2_,*R*_3_ and *R*_4_ with equal weight of one. Then the contig adjacency graph constructed by *S*_1_,*S*_2_,*S*_3_ and *S*_4_ is shown in Fig. 2. Third, we apply a perfect matching program Blossom V [19], whose running time is $\mathcal {O}(n^{4})$, to the contig adjacency graph *G* for finding a perfect matching *M* with maximum weight, where a *perfect matching* is a subset of edges such that each node in the graph is incident to exactly one edge in the subset. Note that if there are multiple perfect matchings with maximum weight in the contig adjacency graph *G*, then we choose one arbitrarily. Finally, we order and orient the contigs of the target genome into scaffolds according to the edge connections in *M* ^′^, where by letting *C*={(*c*_*t*_,*c*_*h*_)|*c*∈*T*}, *M* ^′^ is a subset of *M* obtained by removing some edges with minimum total weight (i.e., with the fewest support from reference genomes) from *M* such that *C*∪*M* ^′^ does not contain any cycles. For instance, consider the contig adjacency graph constructed in Fig. 2. It is not hard to see that *M*={(1_*t*_,4_*h*_),(1_*h*_,2_*t*_),(2_*h*_,3_*t*_),(3_*h*_,4_*t*_)} is a maximum weighted perfect matching in this contig adjacency graph. By removing the edge (1_*t*_,4_*h*_) with minimum weight from *M*, we have *M* ^′^={(1_*h*_,2_*t*_),(2_*h*_,3_*t*_),(3_*h*_,4_*t*_)} and *C*∪*M* ^′^ contains no cycles. As a result, we can obtain a scaffold (+1,+2,+3,+4) from *M* ^′^ for the target genome *T*={1,2,3,4}.

### Usage of Multi-CAR

Multi-CAR is now available online at http://genome.cs.nthu.edu.tw/Multi-CAR/ with a user interface that is intuitive and easy to operate. It takes as input a set of contigs of a target chromosome in multi-FASTA format and one or more reference chromosomes in FASTA format. Meanwhile, the user can assign a weight (positive real number) to each reference chromosome, where the weight reflects the phylogenetic closeness between the target and reference genomes. Basically, the larger the phylogenetic distance, the smaller the weight. In fact, the user can use the default weight of 1 for each reference chromosome if its phylogenetic relationship to the target chromosome is not clear to the user. In addition, it requires the user to choose either “nucleotides” (default) or “translated amino acids” for our Multi-CAR to identify conserved genetic markers between the target and reference chromosomes, which are then utilized by the rearrangement-based algorithm in Multi-CAR to order and orient the contigs of the target chromosome. In the output page, Multi-CAR shows its contig scaffolding results, including total running time, a set of scaffolds and its corresponding multi-FASTA file, dot-plot graphs between the scaffolds of the target chromosome and the reference chromosome, and comparison of dot-plot graphs between before and after contig scaffolding. Basically, for the size of prokaryotic chromosomes, Multi-CAR can finish its contig scaffolding job in several seconds up to a couple of minutes. As to larger chromosomes, the user can choose to run Multi-CAR in a batch mode by providing an email address (optional), via which Multi-CAR can return its scaffolding result to the user when it finishes its job later.

## Results and discussion

### Testing dataset

For validation, we used a real dataset composed of several prokaryotic draft genomes to test Multi-CAR and compared its performance to Ragout [16] and MeDuSa [17] in terms of sensitivity, precision, genome coverage, scaffold number, scaffold N50 size and running time. This real dataset was prepared by Dias et al. [13], containing 19 draft genomes of phylogenetically diverse prokaryotes. Four among these 19 prokaryotic draft genomes have two chromosomes and the others have only one, thus giving a total of 23 chromosomes in this testing dataset (see Table 1). The draft sequences of each such chromosome was then considered as a target and processed separately by each contig scaffolding tool. In this process, we also adopted 20 completely sequenced chromosomes (excluding the target chromosome itself) to serve as the references. These references were chosen by Dias et al. [13] from phylogenetically related prokaryotes deposited in the NCBI database.

**Table 1:** Draft chromosomes used in the testing dataset

Organism	Accession No.	Size (bp)	#CON	COV (%)
*Aciduliprofundum boonei* T469	NC_013926	1,486,778	35	98.63
*Bacillus subtilis* 168	NC_000964	4,215,606	5	99.97
*Bifidobacterium longum* DJO10A	NC_010816	2,375,792	58	85.47
*Brucella melitensis* bv 1 16M (I)	NC_003317	2,117,144	41	90.83
*Brucella melitensis* bv 1 16M (II)	NC_003318	1,177,787	12	99.77
*Brucella pinnipedialis* B2 94 (I)	NC_015857	2,138,342	55	87.47
*Brucella pinnipedialis* B2 94 (II)	NC_015858	1,260,926	34	84.38
*Burkholderia thailandensis* E264 (II)	NC_007650	2,914,771	15	70.34
*Burkholderia thailandensis* E264 (I)	NC_007651	3,809,201	28	89.90
*Chlamydia muridarum* Nigg	NC_002620	1,072,950	4	99.09
*Clostridium cellulovorans* 743B	NC_014393	5,262,222	297	96.54
*Corynebacterium aurimucosum* ATCC 700975	NC_012590	2,790,189	90	92.94
*Corynebacterium efficiens* YS 314	NC_004369	3,147,090	118	95.09
*Micrococcus luteus* NCTC 2665	NC_012803	2,501,097	126	86.25
*Mycobacterium tuberculosis* H37Ra	NC_009525	4,419,977	220	76.84
*Mycoplasma genitalium* G37	NC_000908	580,076	24	78.54
*Saccharopolyspora erythraea* NRRL 2338	NC_009142	8,212,805	238	97.10
*Selenomonas sputigena* ATCC 35185	NC_015437	2,568,361	53	94.01
*Stigmatella aurantiaca* DW4 3 1	NC_014623	10,260,756	470	99.05
*Streptococcus pneumoniae* TIGR4	NC_003028	2,160,842	209	90.31
*Vibrio* Ex25 (I)	NC_013456	3,259,580	176	91.43
*Vibrio* Ex25 (II)	NC_013457	1,829,445	33	95.31
*Yersinia pestis* Nepal516	NC_008149	4,534,590	17	83.86

Column “#CON” contains the number of contigs selected for contig scaffolding experiments by excluding, for example, those contigs not mapped to reference chromosome. Column “COV” gives the fraction of each chromosome covered by selected contigs

In our experiments on this real prokaryotic dataset, we randomly shuffled the input orders of the contigs and the reference chromosomes for each target to eliminate the potential effect of their relative orders on scaffolding results. Moreover, according to the randomly shuffled order of the 20 reference chromosomes, we tested each contig scaffolding tool on the target chromosome by using the first *k* reference chromosomes with *k* varying from 1 to 20. This test was repeated 10 times for each target chromosome, with each time randomly varying the relative order of the 20 reference chromosomes, because the relative order of the references was able to influence the scaffolding results. Next, the evaluation metrics to measure the quality of the scaffolding results returned from these 10 different runs were averaged. Finally, such evaluation metrics obtained from the 23 target chromosomes were further averaged and used for comparing the accuracy performance of all the contig scaffolding tools. In fact, all the draft genomes in our testing dataset are already finished completely and also available from the NCBI database. Therefore, we can utilize these completely finished sequences to derive a *reference order* for the contigs in each draft genome to serve as the standard of truth in our evaluation. Basically, this reference order was derived by mapping all the contigs to their corresponding complete genome and placing them on the positions where they gained the most matches. Moreover, for those contigs that were not matched at all, they were excluded in the reference order.

### Comparisons on sensitivity and precision

Basically, the main quality measure for a scaffolding result is the number of correct contig joins. A join of two contigs in a scaffold is said to be *correct* if they appear consecutively in the reference order (i.e., no other contig in between) and also in the correct orientation. Given the scaffolds of a target chromosome returned by a contig scaffolding tool, we call the number of their correct contig joins as *true positive* (denoted by *TP*) and the number of the others as *false positive* (denoted by *FP*). The *sensitivity* of the scaffolding tool is then defined as *TP*/*P* and its *precision* as *TP*/(*TP*+*FP*), where *P* denotes the number of all contig joins in the reference order. In the following, we compare the performance of Multi-CAR, MeDuSa and Ragout in terms of average sensitivity and precision.

**Fig. 3:**
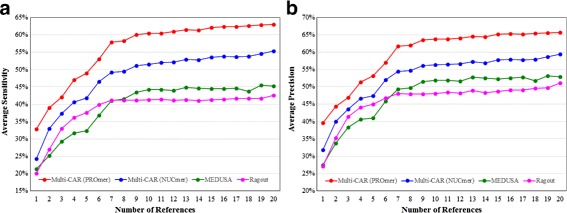
Performance variation of **a** average sensitivity and **b** average precision with respect to the number of reference genomes

In our experiments, we run Multi-CAR (using both NUCmer and PROmer) and MeDuSa (version 1.6) with their default parameters. As for Ragout (version 1.0), however, we run it by using all default parameters, except for utilizing a star tree as the phylogenetic tree and setting the synteny block size to 50, because the phylogenetic tree for each instance was unknown and Ragout returned no or poor results on several instances when the default synteny block sizes (i.e., 5000, 500 and 100) were used. As a result, Fig. 3 a and b show the average sensitivity and precision, respectively, of the three evaluated scaffolding tools over 23 target chromosomes with respect to the increasing number of references from 1 to 20. Clearly, as shown in Fig. 3 a and b, all the three scaffolding tools have an initial rapid improvement on both their average sensitivity and precision (i.e., when the number of references varies from 1 to 7), followed by a much slower performance improvement. In particular, upon using PROmer to identify conserved genetic markers, Multi-CAR gives the best average sensitivity and precision as compared to Multi-CAR running with NUCmer, MeDuSa and Ragout. Note that the reason why Multi-CAR running with PROmer outperforms Multi-CAR running with NUCmer is that PROmer can identify more conserved genetic markers between target and reference genomes to correctly join the contigs than NUCmer, especially when the target and reference genomes are more distantly related. In fact, our Multi-CAR running with NUCmer still performs better than MeDuSa and Ragout in terms of average sensitivity and precision. As for Ragout and MeDuSa, the former has a better performance than the latter in terms of both average sensitivity and precision when the number of the references is between 2 and 7. For the other cases, however, the opposite result that MeDuSa is better than Ragout is observed.

### Comparison on coverage, scaffold number and N50

**Fig. 4:**
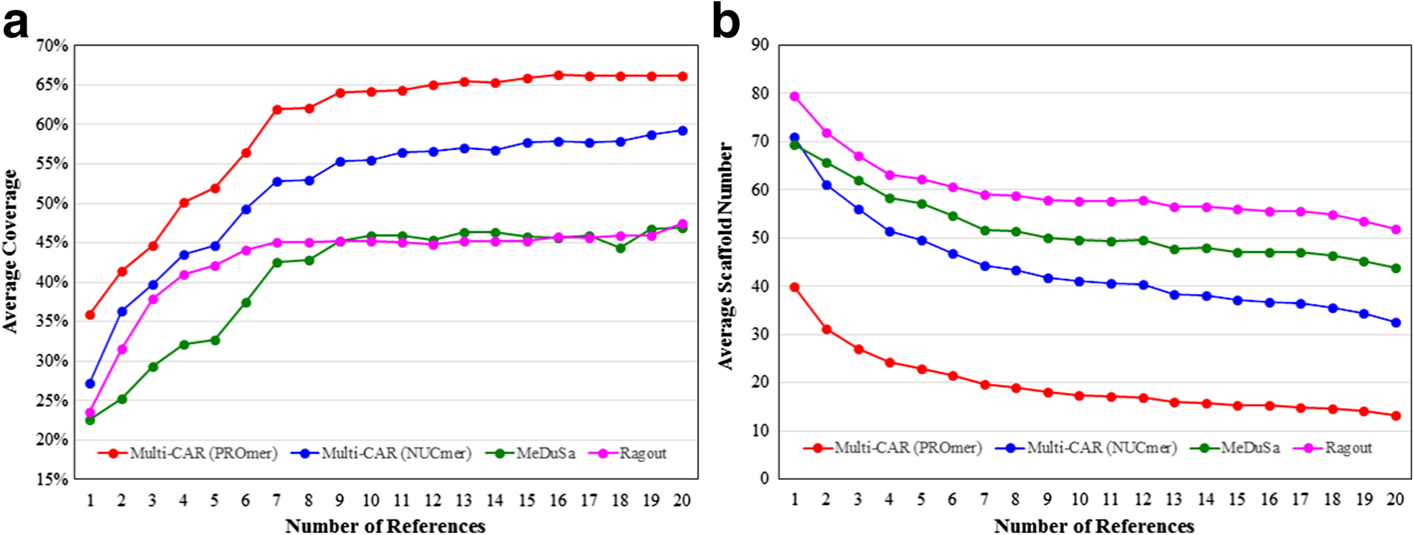
Performance variation of **a** average genome coverage and **b** average scaffold number with respect to the number of reference genomes

Genome coverage is another quality metric to measure how much of the genome being sequenced is actually covered by the scaffolds generated by a contig scaffolding tool [13, 14]. Below, we followed the procedure used in [13, 14] to compute the genome coverage of each scaffolding tool. Basically, a correct contig join in a scaffolding result can be considered as a correct contig adjacency. Given a contig, if its both ends have correct adjacencies, its whole length is thus counted as contributing to the genome coverage. If only one end of this contig has a correct adjacency, its half length is counted. If its both ends has no correct adjacencies, this contig is not considered. The *genome coverage* of a scaffolding result for a target chromosome is then defined as the ratio of the sum of contig lengths that are counted according to the aforementioned rules and the sum of all contig lengths. After an initial rapid improvement, as shown in Fig. 4 a, all the three scaffolding tools reach a somewhat stable average genome coverage. In addition, Multi-CAR running with PROmer (or NUCmer) outperforms MeDuSa and Ragout regarding average genome coverage. On the other hand, Ragout shows a much better performance than MeDuSa in terms of average genome coverage when the number of the references varies between 2 to 8 and for the other cases, their performances are competitive.

**Fig. 5:**
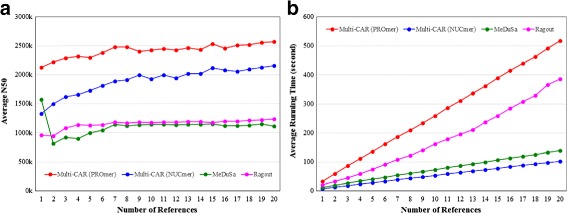
Performance variation of **a** average scaffold N50 size and **b** average running time with respect to the number of reference genomes

Figure 4 b displays the average scaffold number obtained by each scaffolding tool with respect to the increasing number of reference genomes. Clearly, Multi-CAR running with PROmer performs much better than Multi-CAR running with NUCmer, MeDuSa and Ragout, since it produces the fewest average numbers of scaffolds in all cases. In addition, Multi-CAR with NUCmer still has a better performance than MeDuSa and Ragout in almost all cases. In fact, the results of Fig. 4 a and b together suggest that the average scaffold N50 size of Multi-CAR should be longer than those of MeDuSa and Ragout, where the N50 value is defined as the size of the largest scaffold such that 50% of the genome being sequenced is contained in scaffolds of size N50 or larger [20]. As expected, Multi-CAR running with PROmer (and even with NUCmer) indeed performs much better than MeDuSa and Ragout in terms of average scaffold N50 size as shown in Fig. 5 a. As for Ragout and MeDuSa, the average N50 performance of the former is slightly better than that of the latter in almost all cases.

### Comparison on running time

Figure 5 b shows the average running time required by each scaffolding tool to finish its job when the number of reference genomes varies from 1 to 20. Basically, the average running time of each tool increases with respect to the increasing number of the references. As a result, Multi-CAR running with NUCmer performs better than the other tools in terms of required average running time. As mentioned before, however, its average performances on other five metrics (sensitivity, precision, genome coverage, scaffold number and scaffold N50 size) are still inferior to those obtained by Multi-CAR running with PROmer. Although the average running time of Multi-CAR running with PROmer is the longest among all the evaluated scaffolding tools, as shown in Fig. 5 b, it can still finish its scaffolding job in a few up to ten minutes for the size of prokaryotic chromosomes.

## Conclusions

Contig scaffolding is a process of ordering and orienting contigs of a draft genome, which is important and helpful to the finishing of a genome sequencing project. In this study, we introduced a multiple reference-based tool Multi-CAR that can produce more accurate scaffolds of a draft genome based on multiple reference genomes of related organisms. Moreover, it does not require a phylogenetic tree about the draft and reference genomes. In contrast to other similar tools Ragout and MeDuSa, both of which require to solve an NP-hard problem, the algorithm behind our Multi-CAR involves only polynomially solvable problems. By testing on a real dataset composed of several prokaryotic genomes, Multi-CAR exhibited the best average performance in terms of many metrics, such as sensitivity, precision, genome coverage, scaffold number and scaffold N50 size, as compared to Ragout and MeDuSa.
